# Environmental Public Health Tracking of Childhood Asthma Using California Health Interview Survey, Traffic, and Outdoor Air Pollution Data

**DOI:** 10.1289/ehp.10945

**Published:** 2008-04-09

**Authors:** Michelle Wilhelm, Ying-Ying Meng, Rudolph P. Rull, Paul English, John Balmes, Beate Ritz

**Affiliations:** 1 Department of Epidemiology and Center for Occupational and Environmental Health, School of Public Health and; 2 Center for Health Policy Research, School of Public Health, University of California, Los Angeles, California, USA; 3 Northern California Cancer Center, Berkeley, California, USA; 4 Environmental Health Investigations Branch, California Department of Public Health, Richmond, California, USA; 5 Department of Medicine, University of California, San Francisco, California, USA; 6 Division of Environmental Health Sciences, School of Public Health, University of California, Berkeley, California, USA

**Keywords:** air pollution, asthma, children, environmental public health tracking, epidemiology, geographic information system, traffic

## Abstract

**Background:**

Despite extensive evidence that air pollution affects childhood asthma, state-level and national-level tracking of asthma outcomes in relation to air pollution is limited.

**Objectives:**

Our goals were to evaluate the feasibility of linking the 2001 California Health Interview Survey (CHIS), air monitoring, and traffic data; estimate associations between traffic density (TD) or outdoor air pollutant concentrations and childhood asthma morbidity; and evaluate the usefulness of such databases, linkages, and analyses to Environmental Public Health Tracking (EPHT).

**Methods:**

We estimated TD within 500 feet of residential cross-streets of respondents and annual average pollutant concentrations based on monitoring station measurements. We used logistic regression to examine associations with reported asthma symptoms and emergency department (ED) visits/hospitalizations.

**Results:**

Assignment of TD and air pollution exposures for cross-streets was successful for 82% of children with asthma in Los Angeles and San Diego, California, Counties. Children with asthma living in high ozone areas and areas with high concentrations of particulate matter < 10 μm in aerodynamic diameter experienced symptoms more frequently, and those living close to heavy traffic reported more ED visits/hospitalizations. The advantages of the CHIS for asthma EPHT include a large and representative sample, biennial data collection, and ascertainment of important socio-demographic and residential address information. Disadvantages are its cross-sectional design, reliance on parental reports of diagnoses and symptoms, and lack of information on some potential confounders.

**Conclusions:**

Despite limitations, the CHIS provides a useful framework for examining air pollution and childhood asthma morbidity in support of EPHT, especially because later surveys address some noted gaps. We plan to employ CHIS 2003 and 2005 data and novel exposure assessment methods to re-examine the questions raised here.

Asthma is one of the most prevalent chronic conditions affecting children in the United States today. According to National Health Interview Survey data for 2005, > 9 million children < 18 years of age (13%) had ever been diagnosed with asthma (Bloom et al. 2006). The California Health Interview Survey (CHIS) for 2005 indicated a lifetime prevalence of 16% for this age group. Asthma is a multifactorial disease in which genetic susceptibilities influence responses to environmental exposures (Gilmour et al. 2006). Exposure to outdoor air pollution has been widely studied as a potential risk factor for asthma, and it is generally well established that short-term increases can exacerbate respiratory symptoms in children with asthma (Gilmour et al. 2006; Thurston and Bates 2003; Trasande and Thurston 2005). Ozone, particulate matter < 10 and < 2.5 μm in aerodynamic diameter (PM_10_ and PM_2.5_), and nitrogen dioxide are the pollutants linked most consistently with exacerbation of asthma symptoms. Although long-term exposures to O_3_, PM_10_, and NO_2_ have been associated with chronic respiratory impairments such as reduced lung function and growth, bronchitis, and chronic cough, evidence for the impact of air pollution on asthma incidence is less conclusive (Gilmour et al. 2006; McConnell et al. 2002; Trasande and Thurston 2005). Recently, focus has turned to respiratory effects caused by exposure to specific motor vehicle exhaust components such as polycyclic aromatic hydrocarbons adsorbed to particles from diesel engines and ultrafine particles (< 0.1 μm in aerodynamic diameter), which are able to penetrate cellular targets in the lung and enter systemic circulation (Künzli et al. 2003; Li et al. 2002, 2003; Pandya et al. 2002). Various measures of traffic exhaust exposure have been associated with adverse respiratory outcomes, including reduced lung function and growth; asthma hospitalizations; and prevalence of asthma, wheeze, bronchitis, and allergic rhinitis (English et al. 1999; Gauderman et al. 2005, 2007; Kim et al. 2004; McConnell et al. 2006).

Despite the impact of asthma on children’s health, there is no comprehensive system of surveillance at the state or national level for this disease. The national Behavioral Risk Factor Surveillance System (BRFSS) [Centers for Disease Control and Prevention (CDC) 2008a] provides only limited data on asthma prevalence and has poor geographic resolution (i.e., the estimates of asthma prevalence are considered valid only at the state level and thus are not useful for assessing trends in relation to environmental exposures). The National Health Interview Survey (NHIS) (CDC 2008c) and the National Health and Nutrition Examination Survey (CDC 2008b) use a sampling design to represent the population of the entire United States and provide data that cannot be used even at the state level for asthma prevalence estimation; neither of these national surveys yield detailed data on asthma symptoms. Asthma hospitalization data are available from every state, but such data represent only a small fraction of the burden of exacerbations of the disease. Because of the growing body of evidence linking outdoor air pollution exposure to both exacerbation and possibly causation of asthma, tracking its occurrence and severity in relation to pollutant exposures is an important public health goal.

A review by the Pew Environmental Health Commission found that existing efforts to gather information on chronic diseases, such as asthma, and their potential environmental links are highly fragmented and inadequate for truly understanding where, why, and how often these diseases occur; thus, they issued a call to close this environmental health gap (Environmental Health Tracking Project Team 2000; McGeehin et al. 2004). In response, a new initiative to establish a national environmental public health tracking (EPHT) network led by the CDC was launched in 2002. EPHT is the ongoing collection, integration, analysis, and dissemination of data from environmental hazard monitoring, human exposure tracking, and health effect surveillance (McGeehin et al. 2004). As part of this initiative, the goal of the project described here was to develop a model tracking system that links asthma data from the CHIS with existing information on outdoor air pollution exposures, specifically ambient air monitoring station and traffic data. If successful, such linkage and analysis could provide a way to assess impacts of future air pollution control strategies on reducing asthma symptoms in children in California and provide an ongoing mechanism for EPHT of asthma. Here we present results and lessons learned from this first tracking effort based on CHIS 2001 data and ambient air monitoring and traffic data for Southern California.

## Methods

### Data sources

#### Health data

Eligible subjects were individuals 0–17 years of age for whom health data were collected during 2000–2001 as part of the CHIS, who resided in Los Angeles or San Diego County during the same period, and who reported a physician diagnosis of asthma at some point in their lives. The CHIS is a two-stage, geographically stratified, random-digit-dialed telephone survey of California households. One adult was interviewed from each selected household. In households with adolescents and/or children (12–17 and 0–11 years of age, respectively), one adolescent and/or child was randomly selected for an interview. Information on the child respondent was collected from an adult who was most knowledgeable about the child. Except for insurance information provided by the interviewed adult, adolescents were directly interviewed after a parent or guardian gave permission. Information on demographic characteristics, health conditions, health-related behaviors, access to health care, and insurance coverage was collected. Questions pertaining to asthma were modified from existing national health surveys (NHIS and BRFSS), with additional assessment of symptom frequency in children with asthma. Respondents were also asked to report the name of their residential street and the nearest cross-street. Detailed descriptions of CHIS 2001 sampling and survey methods are reported elsewhere (Center for Health Policy Research 2002). This research was approved by the University of California Los Angeles Office for the Protection of Research Subjects and informed consent was obtained from all CHIS 2001 participants.

Within households in Los Angeles and San Diego Counties, interviews were completed for 1,391 adolescents and 3,405 children. We selected 612 respondents (12.8% of those interviewed) who reported ever having been diagnosed with asthma by a physician. Respondents who reported a lifetime diagnosis of asthma were asked to report the frequency of asthma symptoms such as coughing, wheezing, shortness of breath, chest tightness, or phlegm during the 12 months preceding the interview date. In addition, respondents were asked whether they had ever visited a hospital emergency department (ED) or had been hospitalized because of asthma during this period.

#### Exposure data

Exposure to outdoor air pollution was assessed using two sources of existing information: *a*) routine measurement data collected by the California Air Resources Board and South Coast Air Quality Management District at an existing network of air monitors during 1999–2001, and *b*) annual average daily traffic (AADT) data from the California Department of Transportation (Caltrans) for the year 2000. Detailed information on the Caltrans AADT data was previously reported (English et al. 1999; Gunier et al. 2003; Reynolds et al. 2004; Wilhelm and Ritz 2003). Briefly, the AADT represents the annual average number of vehicles per day traveling in both directions along a given road segment based on rotating traffic counts conducted every 3 years. During noncount years, the AADT is estimated using traffic trends for that location. Counts are collected for all highways and most major roads in the state, but counts on smaller, residential roads with low traffic volume are not typically taken.

### Linkage

We estimated traffic density (TD) for each subject based on their reported street of residence and nearest cross-street. Specifically, reported residential cross-streets were geocoded using geographic information system (GIS) software and data (ArcView StreetMap 2000 version 1.1; ESRI, Redlands, CA). We then identified each subject’s probable home street segment, which had the reported nearest intersection at the center bound on both ends by the adjacent cross-streets (Meng et al. 2007). We drew a 500-foot buffer around the probable home street segment of each subject and identified all roadways within this buffer that had an AADT value based on the year 2000 Caltrans data. The 500-foot criterion is based on environmental measurement data showing that the impact of direct traffic emissions on ambient concentrations becomes insignificant at approximately this distance (Zhou and Levy 2007). However, the buffers used here were > 0.028 mi^2^ in area (as would be the case with a 500-foot radius circle around a specific geocoded home location), because the buffers were defined not by a point but by a probable street segment and its length. On average, the buffers were 0.068 mi^2^ in area (range, 0.031–0.199 mi^2^). There was not much difference in buffer size between urban areas (average, 0.067 mi^2^; range, 0.033–0.168 mi^2^) and suburban areas (average, 0.069 mi^2^; range, 0.031–0.199 mi^2^). Most subjects resided in urban areas (71%); 29% were suburban, and none lived in rural areas.

Similar to methods of Gunier et al. (2003) and Reynolds et al. (2004), we estimated the TD value for each subject by first calculating the vehicle miles traveled (VMT) for each attributed road segment within the buffered area; VMT was estimated by multiplying the AADT by the road segment length. We then calculated TD as the sum of the VMT for all road segments in the buffer divided by the area of the buffer, that is,


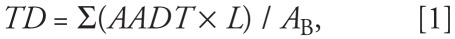


where *TD* is traffic density (vehicles × miles/day/mi^2^), *AADT* is the annual average daily traffic count (vehicles/day), *L* is the length of roadway segment (miles), and *A*_B_ is the area of the 500-foot buffer around the probable street segment (square miles). Subjects with no Caltrans-counted streets within their buffers (*n* = 47) were included in the low-traffic referent category, because we assumed that these individuals had only residential streets with low AADT near their homes.

In addition to TD values, we assigned to each subject the annual average concentrations of O_3_, NO_2_, PM_2.5_, PM_10_, and carbon monoxide measured at the nearest monitoring station within 5 miles of the reported residential cross-street intersection (82% of subjects). We used ArcView GIS (ESRI) software to estimate the distance between each respondent’s residential cross-street intersection or ZIP code population-weighted centroid (for the 18% of subjects whose reported cross-street could not be geocoded) and the nearest station that measured each pollutant. Subjects residing outside a 5-mile range were excluded from the pollutant analyses. Annual average concentrations were calculated for the 1-year period before the interview date. These averages were based on hourly measurements for the gaseous pollutants (CO, NO_2_, and O_3_) and 24-hr average measurements for PM_10_ and PM_2.5_ (with most stations recording measurements every 6 and 3 days for these pollutants, respectively). The 5-mile range was chosen to ensure a large enough radius for sufficient sample size balanced against a possible increase in exposure misclassification due to greater residential distances from monitoring stations.

### Statistical analyses of exposure–asthma outcome relationships

We evaluated associations between air pollution and TD and asthma morbidity using logistic regression. Specifically, we examined differences in our exposure metrics for *a*) children with asthma reporting daily or weekly symptoms in the previous year versus those reporting less than weekly symptoms, and *b*) children with asthma reporting at least one asthma-related ED visit or hospitalization in the previous year versus children with asthma not reporting such visits. The analyses incorporated sampling weights that adjusted for unequal probabilities of selection into the CHIS sample. We initially grouped TD values into quintile categories according to their distributions in the total population. Because the effect estimates in the three middle quintiles were similar, we collapsed them and thus created three categories roughly based on these quintile distributions: *a*) low TD, defined as ≤ 20,000 daily VMT/mi^2^ (approximately ≤ 20th percentile); *b*) medium TD, defined as 20,001–200,000 daily VMT/mi^2^ (approximately 21–80th percentile); *c*) high TD defined as > 200,000 daily VMT/mi^2^ (approximately the 80th percentile).

Measured air pollutants (O_3_, PM_10_, PM_2.5,_ CO, NO_2_) were evaluated continuously, using both single and multipollutant models. We evaluated changes in point and 95% confidence interval (CI) estimates when including covariates such as age, sex, race/ethnicity, poverty level, insurance status, delays in receiving care for asthma, asthma medication use, and county in our models (Table 1). Based on a 10% change-in-estimate criterion (Rothman and Greenland 1998), race/ethnicity and poverty level were included in our final models. Some subjects were excluded because of missing data for these covariates. Final sample sizes for each model are reported in the tables.

## Results

### Data sources

#### Health data

Of the 612 children in Los Angeles and San Diego Counties participating in CHIS 2001 and reporting a physician asthma diagnosis, 56 (9.3%) reported suffering from daily or weekly symptoms, and 68 (11.2%) reported an ED visit or hospitalization for asthma in the previous 12 months. The prevalence of daily or weekly symptoms tended to increase with age, with the highest prevalence observed in children 12–17 years of age with asthma, whereas ED visits and hospitalizations were more prevalent in children ≤ 5 years of age (Table 1). Daily/weekly symptoms were more common in boys than girls. African Americans and Asians/others were more likely to be reported as suffering from daily/weekly symptoms, whereas African Americans and Latinos had higher rates of ED visits/hospitalizations than other racial/ethnic groups (Table 1). Children from a family with an income below the poverty level, experiencing delays in receiving care for asthma, taking asthma medication, and residing in Los Angeles County reported poorly controlled asthma more often than their counterparts without these characteristics (Table 1).

#### Exposure data

In Los Angeles County, 14 stations measured CO, 15 stations NO_2_ and O_3,_ 8 stations PM_10_, and 10 stations PM_2.5_ during 1999–2001. In San Diego County, four stations measured CO, eight stations NO_2,_ nine stations O_3_, six stations PM_10_, and five stations PM_2.5_. On average 8,314, 8,148, and 8,248 hourly air pollution values were available to estimate annual averages for CO, NO_2_, and O_3_, respectively. For PM_10_ and PM_2.5_, 75 and 167 24-hr values were available to estimate annual averages, respectively. Annual average concentrations of NO_2_, PM_2.5_, and PM_10_ were highly positively correlated with each other and moderately correlated with CO (Table 2). Levels of O_3_ were strongly negatively correlated with these pollutants, whereas residential TD measures were not correlated with any of the station-based pollutant measures.

### Linkage

We were able to successfully geocode reported residential cross-street intersections and calculate TD values for 500 (81.7% of 612) respondents. Only three subjects who were geocoded could not be assigned a TD value because of missing AADT data in the Caltrans file. The reasons reported locations were not successfully mapped included missing information for one or both streets (63%) and inability to find a reported cross-street in the reference street map (i.e., one or both streets were not found based on spelling provided or reported streets did not intersect, 37%). The mean TD value was 152,311 (median of 86,513), with a range of 0–1,557,027 daily VMT/mi^2^ (Table 2). The percentage of subjects excluded from analyses based on annual average air pollution averages varied depending on pollutant (because not every station measured every pollutant, and we used a 5-mile exclusion criterion, as explained above). Approximately 26%, 47%, 36% of subjects were excluded from the O_3_, PM_10_, and PM_2.5_ analyses, respectively. Mean annual average pollutant concentrations did not differ substantially for subjects included versus excluded from analyses based on this criterion; however, the percentages of children with asthma reporting daily or weekly symptoms or ED visits/hospitalizations were lower in the excluded population (by approximately 2–5% depending on pollutant and outcome).

### Statistical analyses of exposure–asthma outcome relationships

Based on logistic regression models, we did not observe associations between our asthma symptom outcome measures and NO_2_ or CO; thus, we limit the following discussion to residential TD, O_3_, and particles (PM_10_ and PM_2.5_) (Tables 3 and 4). We observed an approximately 2-fold increase in the odds of daily/weekly symptoms in children with asthma for each 1 part per hundred million (pphm) increase in annual average O_3_ [odds ratio (OR) = 1.96; 95% CI, 1.23–3.13], and this estimate did not change appreciably when we controlled for race/ethnicity and poverty level or when we added particle measures to the model, although the 95% CIs widened because of the inclusion of these covariates and the reduction in sample size (Table 3). There were suggestive associations between particles (PM_10_ and PM_2.5_) and daily/weekly symptoms after adjustment for O_3_, but estimates were imprecise. We also observed an approximately 2-fold increase in the odds of ED visits/hospitalizations for asthma per 1-pphm increase in O_3_ after adjusting for particles (particle and O_3_ exposure estimates were strongly negatively correlated). Furthermore, we also estimated 2- to 3-fold increases in the odds of ED visits/hospitalizations per 10-μg/m^3^ increases in PM_10_ and PM_2.5_ after adjusting for O_3_ (Table 3).

We observed associations between residential proximity to traffic and asthma-related ED/hospitalizations but not for daily/weekly symptoms (Table 4). We estimated an approximately 3-fold increase in the odds of ED visits/hospitalizations for children with asthma in the highest TD exposure category (TD > 200,000 daily VMT/mi^2^) compared with those with TD values ≤ 20,000 daily VMT/mi^2^ after adjusting for race/ethnicity and poverty level (OR = 3.27; 95% CI, 1.08–9.89). A model that included TD, O_3_, and particle measures was consistent with these findings (results not shown). Approximately 48% of the subjects with TD > 200,000 daily VMT/mi^2^ had a freeway within their buffer. (Approximately 41% of children in Los Angeles County versus ~ 84% in San Diego County had a freeway in their buffer, yet of all children with TD values > 200,000, ~82% resided in Los Angeles.)

## Discussion

This project demonstrated methods for linking and analyzing existing health and environmental databases as part of the national EPHT initiative currently under development in the United States (McGeehin et al. 2004). Based on this linkage/analysis effort, we documented that children with asthma living in more highly polluted areas—as assessed by ambient air monitoring data and measures of traffic near homes—experienced worse asthma morbidity than those living in less polluted areas. Our findings are in general agreement with existing evidence that short-and long-term exposure to O_3_ and particulate matter increases childhood asthma morbidity (Gilmour et al. 2006; Trasande and Thurston 2005) and previous reports that children with asthma residing near heavy traffic are more likely to seek medical attention or be hospitalized for asthma than those residing near low-traffic roadways (Edwards et al. 1994; English et al. 1999; Lin et al. 2002). More important, however, our goal was to evaluate the overall success of this project in terms of an EPHT framework. In the following sections, we discuss the advantages and disadvantages of the data sources, linkage, and analyses presented here for EPHT of air pollution and childhood asthma morbidity.

### Data sources

#### Health data

The CHIS is the largest statewide health survey in the country. CHIS 2001 included 18,393 children (ages 0–17 years) compared with 13,376 children included in the 2000 NHIS. Because of the sampling design, CHIS data can provide disease prevalence estimates at the local level (e.g., county level) and for specific racial/ethnic groups (the survey is conducted in six languages). These factors, along with the biennial data collection, are all strengths within the context of EPHT. Another major strength is collection of residential information at the cross-street (CHIS 2001 for Los Angeles and San Diego Counties) and residential address level (CHIS 2003 and 2005 for all counties), which allows improved spatial resolution for air pollution exposure assessment.

Limitations of the CHIS asthma data include their cross-sectional nature and reliance on self-report of physician diagnoses. As with all cross-sectional data, there is potential temporal ambiguity between exposure and disease. In this study, we did not have lifetime residential histories, and we assigned monitoring stations and estimated TD based on current home location. Thus, our estimates could be biased depending on residential mobility patterns of children with asthma. If families with children with poorly controlled asthma tended to move away from highly polluted areas after learning that air pollution might worsen asthma, our pollution associations may be underestimated. Subsequent CHIS surveys collect information on timing of asthma diagnoses and length of residence in the same house and neighborhood. Thus, in future analyses, we will be able to examine the extent to which this bias may have affected our results. In general, collection of information on residential and school histories would help improve exposure assessment further and allow a better quantification of associations between air pollution and asthma morbidity in children as part of EPHT efforts (as discussed below). CHIS 2001 relied on parental or self-reports of physician-diagnosed asthma and related symptoms and thus may have missed a certain segment of the population with undiagnosed asthma. CHIS 2003 asked about symptoms among individuals who were not physician diagnosed. Finally, the CHIS is a telephone-based survey and generally achieves a response rate of 40%. Estimating the magnitude and direction of bias due to nonresponse on our study results would require additional data collection (i.e., a follow-up study of a sample of nonresponders to determine health and air pollution exposure status). A discussion of other potential health data sources for tracking childhood asthma and its relation to air pollution is provided in Supplemental Material (online at http://www.ehponline.org/members/2008/10945/suppl.pdf).

#### Exposure data

The advantages of using existing government air monitoring data to assess air pollution exposures for EPHT are that the data are readily available and, in general, measurements have been consistently recorded over many years and offer a fine temporal resolution (i.e., hourly, daily, or every third or sixth day measurements are available). The tradeoff for this fine temporal resolution is lower spatial coverage, that is, the number of stations available to characterize concentration gradients throughout an urban area is limited. Such data may be inadequate for accurate exposure assessment, especially for pollutants like CO, nitrogen oxides (NO_x_), and ultrafine particles that exhibit small-scale spatial variability in concentrations depending on proximity to sources (Zhou and Levy 2007). Another major issue with regard to exposure assessment is accounting for interindividual exposure variability due to personal mobility and time spent outdoors versus indoors.

Epidemiologic studies are increasingly using residential and/or school proximity to traffic as surrogate measures of motor vehicle exhaust exposure (Salam et al. 2008), as we did in this study. Caltrans traffic data are a readily available source of information for generating such measures (see the Traffic Density Mapping Tool of the California Environmental Health Tracking Program; www.ehib.com/tools), but there are a number of limitations involved (Wilhelm and Ritz 2003). Briefly, these shortcomings include *a*) lack of information on vehicle engine types (gasoline vs. diesel) and vehicle ages in a given 24-hr traffic count, which are important determinants of emission levels, *b*) lack of information concerning the influence of meteorology (e.g., wind direction) on dispersion of these emissions, and *c*) differences between outdoor and indoor exhaust concentrations based on ventilation characteristics of the home. However, as noted previously, a growing number of studies are linking relatively simple measures of traffic exposure such as the one we used in this study to adverse respiratory outcomes in children (English et al. 1999; Gauderman et al. 2005, 2007; McConnell et al. 2006). For example, Gauderman et al. (2005) reported that a relatively simple measure of residential distance to freeway was as strongly and precisely associated with childhood asthma as a more complex exposure estimate based on air dispersion modeling of freeway emissions and even measured NO_2_ concentrations. However, whether this would be the case in other settings is currently unknown; very few studies have used more complex models that account for time–activity patterns of children and spatial heterogeneity of air pollution concentrations within urban areas. Alternatives for air pollution exposure assessment include geostatistical methods such as kriging (e.g., Jerrett et al. 2005; Kunzli et al. 2005) and land use–based regression modeling (e.g., Jerrett et al. 2007; Ross et al. 2006), as discussed below.

### Linkage

An advantage of the CHIS for linkage of health and environmental data is knowledge of residential locations at the cross-street (CHIS 2001) and address (CHIS 2003 and 2005) level. Knowledge of residential locations is particularly important for assessing source-specific exposures, such as exposures to certain key traffic exhaust pollutants (e.g., ultrafine particles) whose concentrations change rapidly within approximately 300 m of the source (roadway) (Zhou and Levy 2007). Mapping home locations to the census tract or even census block group level likely results in exposure misclassification for such pollutants. Here, only residential cross streets were available, which likely introduced error into the traffic exposure metric; future CHIS surveys collect information on residential addresses. Knowledge of residential locations at the cross-street and address level also allows determination of distance to existing monitoring stations; thus, subjects living far from stations can be excluded from analyses (based on the assumption that accuracy of exposure assessment decreases as one moves farther away from a station). The disadvantages of using such exclusion criteria are that they reduce sample size and may limit generalizability.

Geostatistical methods such as kriging and land use–based regression modeling are alternatives for assessing air pollution exposure [see Jerrett et al. (2005) for an in-depth discussion of these methods and others such as inverse distance weighting and air dispersion modeling]. Kriging spatially interpolates pollution levels measured at existing monitoring stations across urban areas. A major advantage is that it allows the estimation of both predicted values and their standard errors (kriging variance) at unmeasured locations (Jerrett et al. 2005). However, the predictive capability of this method is dependent on the density of monitoring stations in a given area. Reliance on existing monitors (which are generally limited in number) normally results in surfaces that oversmooth the true pattern of pollution and may introduce large errors in estimates over portions of a study area for which few observations are available (Jerrett et al. 2005). This problem may be more severe for pollutants known to vary significantly over small scales, such as NO_x_ and other direct traffic pollutants. For these pollutants, land use–based regression (LUR) detects small-area variations in air pollution more effectively than kriging (Briggs 1997; Lebret et al. 2000). In the LUR modeling approach, concentrations of vehicle exhaust markers such as NO_x_ are measured simultaneously at many locations throughout an urban area, using relatively inexpensive passive monitors (e.g., Ogawa monitors). Various GIS parameters (such as traffic and roadway density, population density, and land use) are used to predict the measured concentrations (Jerrett et al. 2007). The developed model can then be used to estimate concentrations at home and school locations of study subjects based on GIS parameter values at these locations. Although these methods allow generation of air pollution exposure estimates at a wide number of locations, a number of statistical assumptions are needed (Jerrett et al. 2005). They also require additional expertise to implement, and in the case of LUR, additional data collection, which can be labor intensive. Furthermore, LUR models developed in one area may not be readily transferable to other urban areas.

Another major issue in air pollution exposure assessment is accounting for time–activity patterns of the subjects (time spent at home, at school, in vehicles) and extent of infiltration of outdoor air pollution into indoor spaces (homes and schools). Exposure misclassification resulting from the lack of such information may be one explanation for the differences we observed in particle and TD results for the two outcomes (daily/weekly symptoms vs. ED visits/hospitalizations) in this study. Specifically, the particle associations for daily/weekly symptoms were weaker than for ED visits/hospitalizations. This may reflect more nondifferential exposure misclassification of our residence-based particle measures for older children more likely to spend time away from home during the day; although daily/weekly symptoms were more frequently reported for this age group, ED visits/hospitalizations were more common in younger children (Table 1). The same explanation could also hold for the null association we observed between residential TD and daily/weekly symptoms. Although levels of O_3_ tend to be more spatially homogeneous within communities (Avol et al. 1998; Geyh et al. 2000; Monn 2001), several O_3_ exposure assessment studies in Southern California children have noted the important contribution of time–activity patterns (e.g., time spent outdoors) and housing characteristics (e.g., use of air conditioning, age and size of house, use of open windows and fans for ventilation) to personal O_3_ levels (Avol et al. 1998; Geyh et al. 2000; Liu et al. 1997; Xue et al. 2005). In this study, we observed associations between ambient O_3_ measures and both outcomes, but point estimates were generally lower for ED visits/hospitalizations (younger children). This may suggest that station-based measures of O_3_ are more accurate estimates of exposure for older children, who may spend more time outdoors and who may engage in outdoor activities that increase their exposure, compared with younger children (infants and young toddlers), who may spend more time indoors and thus generally have lower O_3_ exposures. Subsequent CHIS surveys collected information on school locations so that potential differences in exposure based on location of the children can be taken into account and the impact on estimates of association can be assessed. Increased sample sizes when adding CHIS 2003 and 2005 data will also allow us to determine the robustness of our findings, which were relatively imprecise when we relied on 1 year of data for Los Angeles and San Diego Counties only. Future EPHT tracking efforts focusing on O_3_-related health effects may also be improved by collecting housing information, such as air conditioning use and house size, and age and time–activity information, such as time spent at school and outdoors. Collection of such data, in concert with more spatially resolved outdoor pollution gradients (e.g., through kriging or LUR), would allow the development of more complex exposure models for air pollution (see, for example, Marshall et al. 2006 and Wu et al. 2005). This type of modeling may also be possible without the collection of time–activity and housing information directly from subjects as part of surveys, that is, by using data from the U.S. Census and existing travel demand surveys coupled with stochastic modeling. [See Marshall et al. (2006) for an example using existing Southern California Association of Governments travel demand and mobility data] However, the usefulness of such stochastic modeling for EPHT needs further investigation.

### Statistical analyses of exposure–asthma outcome relationships

The CHIS includes a large, statewide sample, thereby facilitating statistical analyses of associations between air pollution and asthma while taking into consideration potential confounding factors. For this particular demonstration project, residential cross-street information was available only for respondents in Los Angeles and San Diego Counties; thus, our resulting air pollution effect estimates were relatively imprecise. CHIS 2003 and 2005 each included approximately 3,000 children with a reported asthma diagnosis or asthma-like symptoms and residential address information, providing much larger sample sizes for future analyses. In addition to a relatively large sample size, CHIS data include information on a number of potential confounding factors including race/ethnicity, income, health insurance status, usual source of care, type of usual source of care, experience of delays in receiving asthma care, and asthma medication use. For this study, we lacked information on some potentially important confounders of the association between air pollution and asthma morbidity, including exposure to secondhand tobacco smoke and indoor allergens such as pets, cockroaches, and molds, parental history of asthma, day care attendance, and breast-feeding history. Such factors may also be important to investigate as effect measure modifiers of the relationship between air pollution exposure and asthma exacerbation. Subsequent CHIS surveys (CHIS 2003 and 2005) will allow us to examine potential residual confounding of our outdoor air pollution effect estimates by some of these factors. Systematic collection of all of these potential confounding variables may not be feasible within the framework of EPHT. Nevertheless, within an EPHT framework it may be necessary to first establish a minimally sufficient set of variables necessary to allow estimation with little residual confounding.

The goals of EPHT are not only to quantify and examine links between exposure to environmental agents—such as air pollution—and disease, but also to determine whether increases or decreases in exposure result in subsequent changes in health indicators. From a public health perspective, such information supplies data to support policy decisions—for example, mandating cleaner-burning motor vehicles and fuels. Previous studies have capitalized on natural experiments (Friedman et al. 2001; Pope 1996) or prospectively followed schoolchildren (Avol et al. 2001; McConnell et al. 2003) to demonstrate a lowering of asthma morbidity resulting from changes (reductions) in air pollution. However, serial cross-sectional surveys such as the CHIS may be more amenable to EPHT for assessing the impact of air pollution regulations, as they provide asthma prevalence data on a more routine basis and cover a diverse and representative sample of children (in this case, for California). The specific mechanics for relating changes in regulatory reductions in air pollution to consequent changes in asthma health end points are beyond the scope of this paper. We currently have exposure metrics only for the 2001 CHIS participants. However, major factors that would need to be taken into account for such analyses include *a*) disentangling the effects of meteorology versus emissions reductions on air pollution levels, *b*) evaluating and accounting for potential confounding factors that may also change over time (e.g., changes in access to health care, use of asthma management plans) in statistical models, and *c*) attributing emissions reductions and subsequent health improvements to specific regulations. It may also be of interest to examine “what if” scenarios whereby the impacts of projected air pollution levels with and without the implementation of a specific emission reduction program on subsequent health end points are evaluated. This may be particularly important in areas where major changes in population or VMT are occurring.

## Conclusions

We demonstrated a method of linking and analyzing existing health and environmental data as part of the national EPHT initiatives currently under development in the United States. Our data linkage and analyses indicate that children with asthma living in high O_3_ and PM_10_ areas in Los Angeles and San Diego Counties experience symptoms more frequently than those living in less-polluted neighborhoods. Children with asthma living close to heavy traffic also report more ED visits and hospitalizations than those with less traffic near their home. Limited asthma surveillance information other than data from hospital discharge records is collected at the state or national level. The advantages of the CHIS data for EPHT are its relatively large sample size and representation of the diverse California population, biennial data collection including information on potentially important covariates, and residential locations at the address level, allowing for more spatially refined exposure assessment. Disadvantages include the cross-sectional nature of data collection, reliance on parental reports of asthma diagnoses and symptoms, lack of information on some potential confounders, and lack of residential and school histories. Some of these shortcomings were addressed in CHIS 2003 and 2005. Overall, the results from these first linkage efforts indicate the potential importance of collecting routine data on children’s home and school locations (especially for studies interested in traffic exhaust impacts). Additionally, timing of asthma diagnoses, residential histories, and assessment of respiratory symptoms in undiagnosed children may be required to rule out potential biases in EPHT linkage efforts. Collection of information on children’s time–activity patterns (time spent outdoors) and housing characteristics (e.g., use of air conditioning and level of ventilation) and advanced exposure modeling (LUR and kriging) may further improve exposure assessment.

## Figures and Tables

**Table 1:** Weighted prevalence of frequent symptoms or ED visits/hospitalizations by demographic characteristics among CHIS children with reported asthma diagnoses, Los Angeles and San Diego Counties.

	Daily or weekly symptoms (Total children with asthma = 597)		ED visit or hospitalization (Total children with asthma = 607)	
Characteristic	Prevalence (%)	No. total population	Prevalence (%)	No. total population
Age (years)				
0–5	4.2	117	19.8	119
6–11	7.1	257	11.8	263
12–17	18.5	223	7.9	225
Sex				
Male	12.6	341	11.2	346
Female	8.6	256	12.9	261
Race/ethnicity				
Latino	9.6	176	16.1	182
Asian/other	13.4	81	9.1	83
African American	16.6	97	15.6	99
White	8.7	243	7.8	243
Household federal poverty level (%)^a^				
< 100	16.6	107	19.8	109
100–299	9.6	221	11.5	225
≥ 300	8.4	269	7.5	273
County				
Los Angeles	11.1	481	13.7	489
San Diego	9.8	116	6.8	118
Insurance status				
Currently uninsured	23.7	45	18.1	45
Uninsured any time in past 12 months	15.8	25	19.2	25
Insured entire past 12 months	9.1	527	10.9	537
Delays in care^b^				
Yes	27.4	24	37.5	24
No	10.2	571	11.1	580
Taking asthma medication				
Yes	17.6	296	20.4	300
No	4.7	300	4.4	306

a Percentages were defined using 2001 federal poverty guidelines ($9,044 for one person, $11,559 for a family of two; incomes at ≥ 300% federal poverty level were three times these amounts).

b Individuals who reported delaying or foregoing any medical care they felt they needed (such as seeing a doctor, a specialist, or other health professional) for asthma were assigned a value of 1 for delays in care.

**Table 2:** Pearson correlation coefficients for annual average air pollutant concentrations for residents within 5 miles of a monitoring station.

Pollutant	Mean (range)	O_3_	PM_10_	PM_2.5_	NO_2_	CO	TD
O_3_ (pphm)	2.1 (1.1–4.2)	1					
PM_10_ (μg/m3)	37.7 (26.2–46.9)	−0.70	1				
PM_2.5_ (μg/m3)	19.3 (10.6–24.6)	−0.79	0.85	1			
NO_2_ (pphm)	3.0 (1.4–4.4)	−0.82	0.83	0.89	1		
CO (ppm)	1.0 (0.34–1.8)	−0.67	0.41	0.60	0.57	1	
TD (daily VMT/mi^2^)	152,311 (0–1,557,027)^a^	−0.16	0.12	0.16	0.17	0.02	1

a Average includes 47 subjects assigned TD = 0 because of no Caltrans-counted streets in buffer.

**Table 3:** Association [OR (95% CI)] between annual average air pollution concentrations and asthma outcomes in CHIS children 0–17 years of age.

	Daily/weekly symptoms				ED visit or hospitalization			
	Single pollutant only (crude)	Single pollutant, race/ethnicity, poverty level	Two pollutants (O_3_ + PM_10_), race/ethnicity, poverty level	Two pollutants (O_3_ + PM_2.5_), race/ethnicity, poverty level	Single pollutant only (crude)	Single pollutant, race/ethnicity, poverty level	Two pollutants (O_3_ + PM_10_), race/ethnicity, poverty level	Two pollutants (O_3_ + PM_2.5_), race/ethnicity, poverty level
O_3_^a^	44, 391	44, 391	36, 269	36, 327	53, 390	53, 390	38, 272	47, 322
(per 1 pphm)	1.96 (1.23–3.13)	2.09 (1.28–3.41)	2.29 (1.01–5.23)	3.51 (1.45–8.46)	1.16 (0.74–1.81)	1.35 (0.85–2.14)	2.89 (1.32–6.34)	2.48 (1.14–5.38)
PM_10_^a^	36, 269	36, 269	36, 269	—	38, 272	38, 272	38, 272	—
(per 10 μg/m^3^)	1.09 (0.63–1.87)	1.09 (0.62–1.93)	1.82 (0.86–3.87)		1.46 (0.85–2.52)	1.46 (0.84–2.55)	2.76 (1.33–5.71)	
PM_2.5_^a^	36, 327	36, 327	—	36, 327	47, 322	47, 322	—	47, 322
(per 10 μg/m^3^)	0.70 (0.29–1.67)	0.69 (0.27–1.72)		3.89 (0.86–17.5)	1.26 (0.57–2.80)	1.09 (0.47–2.50)		3.68 (0.98–13.8)

a Values represent number of children with asthma with daily/weekly symptoms or ED visits/hospitalizations in previous year, total number of children with asthma.

**Table 4:** Association (OR, 95% CI) between residential TD (in daily VMT/mi^2^) and asthma outcomes in CHIS children 0–17 years of age.

	Daily/weekly symptoms		ED visit or hospitalization	
	TD only (40, 453)^a^	TD, race/ethnicity, poverty level (40, 453)^a^	TD only (52, 448)	TD, race/ethnicity, poverty level (52, 448)^a^
TD ≤20,000	1.00	1.00	1.00	1.00
20,001 < TD ≤200,000	1.12 (0.53–2.34)	0.95 (0.44–2.06)	3.05 (1.11–8.38)	2.45 (0.87–6.88)
TD > 200,000	0.77 (0.30–2.03)	0.61 (0.22–1.69)	5.01 (1.72–14.6)	3.27 (1.08–9.89)

a Values represent number of children with asthma with daily/weekly symptoms or ED visits/hospitalizations in previous year, total number of children with asthma.
